# *Desulfuromonas* sp. 'CSMB_57’, isolation and genomic insights from the most abundant bacterial taxon in eastern Australian coals

**DOI:** 10.1099/mgen.0.000857

**Published:** 2022-08-23

**Authors:** Andrew G. McLeish, Paul Greenfield, David J. Midgley, Ian T. Paulsen

**Affiliations:** ^1^​ Department of Molecular Sciences, Macquarie University, North Ryde, Sydney, Australia; ^2^​ Department of Energy, Commonwealth Scientific and Industrial Research Organisation (CSIRO), Lindfield, Sydney, Australia; ^3^​ Department of Biological Sciences, Macquarie University, North Ryde, Sydney, Australia

**Keywords:** coal bed microbiology, comparative genomics, coal seam microbiology, geomicrobiology

## Abstract

One of the most abundant and ubiquitous taxa observed in eastern Australian coal seams is an uncultured *Desulfuromonas* species and part of the Coal Seam Microbiome dataset assigned as ‘CSMB_57’. Despite this abundance and ubiquity, knowledge about this taxon is limited. The present study aimed to generate an enrichment culture of *Desulfuromonas* sp. ‘CSMB_57’ using culturing strategies that exploit its sulphur-reducing capabilities by utilizing a polysulfide solution in a liquid medium. Using dilution to extinction methods, a highly enriched culture was successfully generated. The full-length 16S rRNA sequence revealed that all closely related taxa were observed in subsurface environments suggesting that *D*. sp. ‘CSMB_57’ may be a subsurface specialist. Subsequently, the DNA from the enrichment culture was sequenced and the genome of *D*. sp. ‘CSMB_57’ was assembled. Genomic annotation revealed a high number of CRISPR arrays for viral defence, a large array of ABC transporters for amino acid and peptide uptake, as well as genes likely associated with syntrophy such as genes associated with type-IVa pilus, often used for direct interspecies electron transfer, and multiple hydrogenases capable of producing hydrogen. From the various genomic observations, a conceptual ecological model was developed that explores its possible syntrophic roles with hydrogenotrophic methanogens and acetogenic bacteria within coal-seam environments.

## Data Summary

All Supplementary Data files used in the analyses are available at https://doi.org/10.25919/fdpb-mf14.

This Whole Genome Shotgun project has been deposited at DDBJ/ENA/GenBank under the accession JAFCIY000000000. The version described in this paper is version JAFCIY020000000.

**Impact Statement**

For the last decade, researchers have sought to understand how fossilized carbon in coal is degraded to methane. Key to this endeavour is understanding the roles key taxa play in coal-seam environments. In Australia, the most widespread and abundant bacterial taxon in subsurface coal seams is a *Desulfuromonas* that is part of the coal-seam microbiome OTU ‘CSMB_57’. Using classical culturing and a key modification of the culture medium using polysulfide, this taxon was brought into a highly enriched culture, the DNA from the culture was sequenced and the genome of *Desulfuromonas* sp. ‘CSMB_57’ was sequenced and analysed. While its growth in sulphur-reducing media reveals it is capable of sulphur reduction, the coal seams of eastern Australia typically contain only trace amounts of inorganic or organic sulphur. Other modes of metabolism must be required for its abundance in these environments. Genomic analysis revealed genes for hydrogen production, type-IV pili, and a range of transporters for scavenging materials from the harsh subsurface environment suggest *D*. sp. ‘CSMB_57’ is likely involved in syntrophies with the dominant methanogen (typically *Methanobacterium* or *Methanocalculus* species) in these environments. A conceptual, ecological model of its likely life-strategy within the coal-seam environment based on its genome is proposed and discussed.

## Introduction

Methane is a key transitional fuel in the shift from coal-fired electricity generation to renewable energy [1–3]. In Australia, methane is expected to form part of the energy mix for the next four decades [4–6] and may play a key role as a source of blue hydrogen through steam methane reforming [7] with the carbon sequestered in a variety of forms. Globally, coal-seam methane reserves have been demonstrated to include significant methane that is microbially generated and, as such, considerable research effort has been directed towards enhancing microbial generation of methane *in situ* (reviewed in [8] and [3]). Improving microbial generation of methane, however, requires a deeper understanding of the roles microbes play in subsurface coal seams. Understanding these processes will also provide insight into the flow of carbon in subsurface environments.

Among the most consistently observed and abundant bacteria from eastern Australian coal seams is a taxon from Desufuromonadaceae, a putative *Desulfuromonas* species, which maps to OTU ‘CSMB_57’ in the coal-seam microbiome (CSMB) reference set [9]. The ubiquity and high abundance of this ‘CSMB_57’ taxon in eastern Australian coal seams was highlighted by [9], which observed the taxon in all 28 spatially and temporally separated coal formation water samples (six samples from three wells in the Bowen Basin, 11 samples from five wells in the Sydney Basin, and 11 samples from five wells in the Surat Basin) with their abundances reported to be very high (~25% Surat, and ~10 % for both the Sydney and Bowen Basins). This taxon was first observed in 2007 in a 16S rRNA survey of a ~1 km subsurface coal seam in Hokkaido, Japan. Researchers from this study noted that the taxon was most closely related to *Syntrophotalea acetylenica* (previously known as *Pelobacter acetylenicus *) using a full-length 16S rRNA sequence [10]. Other *Desulfuromonas* genomes have been sequenced from Australian coal seams. Indeed, the metagenomically assembled genome ‘*Candidatus* Desulfuromonas subbituminosa’ (IMG genome ID: 2603880216), which was observed in Roma, Queensland, was sequenced in 2011 (Robbins and Tyson, unpublished) and 16S rRNA analysis suggests that it is the most closely related taxon to ‘CSMB_57’. This taxon may be conspecific to ‘CSMB_57’ and comparisons between these two taxa would additionally help resolve the taxonomic ambiguity of species within the Desulfuromonadales order.

The phylogenetic inconsistencies within the Desulfuromonadales order has been known for decades [11]. Recently, there has been a major proposal for a thorough reclassification of the Deltaproteobacteria class as a new super-phylum with four new phyla (Desulfobacterota, Myxococcota, Bdellovibrionota and the placeholder SAR324) being introduced [12]. This reclassification has proposed changes to both the *Pelobacter* and *Desulfuromonas* genera. The *Pelobacter* genus does not form a coherent cluster as phylogenetic studies frequently demonstrate the polyphyletic nature of this genus, with different *Pelobacter* species being variously interspersed among other *Desulfuromonas* and *Geobacter* [11–14]. As such, the *Pelobacter* genus was redistributed into three new genera: *Syntrophotalea *, *Pseudopelobacter* and *Seleniibacterium* [12, 14]. There are also considerations for the reclassification of several *Desulfuromonas* species with new genera such as *Pseudodesulfuromonas *, *Trichloromonas* and *Deferrimonas* being recommended [12, 14].

Regardless of their taxonomy, the *Desulfuromonas* genus is widely known for its ability to reduce elemental sulphur (S^0^) to hydrogen sulfide (H_2_S) [15, 16] and Fe (III) oxide reduction with the use of *c*-type cytochromes [17]. It is highly unlikely, however, that they are engaged in this process in eastern Australian coal seams as the environment typically contains only trace amounts of S^0^ and Fe (III) [18]. The ecological role of the ‘CSMB_57’ taxon in the coal seam is thus unclear. Ritter *et al*. [3] suggested that coal-seam Deltaproteobacteria may take on syntrophic roles, though evidence for this with *Desulfuromonas* species is lacking.

The aim of the present study was to isolate and sequence an abundant and ubiquitous bacteria present in eastern Australian coal seams, *Desulfuromonas* sp. ‘CSMB_57’, in order to further our understanding of the role it plays in the subsurface environment.

## Methods

### Source of inoculum

In the present study, microbes were sourced from the Surat Basin, well 5 [9]. The sample used, its methods of collection, water chemistry and microbial community composition have been described in detail previously [9].

### Enrichment media

Five media were used in the present study:

(i) Modified basal sulphur reducing bacteria enrichment medium [15] contained l^−1^ : 1.0 g KH_2_PO_4_, 0.3 g NH_4_Cl, 1.0 g MgSO_4_-7H_2_O, 2.0 g MgCl_2_-6H_2_O, 2.0 g NaCl, 0.1 g CaCl_2_-2H_2_O, 3.0 g NaHCO_3_, 2 ml SL-10 trace element solution [19], 2 ml of a 2M solution of H_2_SO_4_, 20 µg biotin, 20 µg vitamin B12, 0.5 g sodium acetate, and 3 ml polysulfide solution. The medium was degassed prior to the addition of 0.3 g l^−1^ Na_2_S under anoxic conditions (headspace 95% Ar, 5 % H_2_) and pH was adjusted to 7.8. The SL-10 trace element solution contained l^−1^ : 10 ml HCl (25 %), 1.5 g FeCl_2_-4H_2_O, 70 mg ZnCl_2_, 100 mg MnCl_2_-4H_2_O, 6 mg H_3_BO_3_, 190 mg CoCl_2_-6H_2_O, 2 mg CuCl_2_-2H_2_O, 24 mg NiCl_2_-6H_2_O, and 36 mg Na_2_MoO_4_-2H_2_O. The polysulfide solution contained 10 g Na_2_S-9H_2_O and 3 g elemental sulphur in 15 ml of distilled water, which was autoclaved at 121 °C for 30 min.

(ii) Modified Baars sulfate enrichment medium [20] contained l^−1^ : 0.5 g K_2_HPO_4_, 1.0 g NH_4_Cl, 1.0 g CaCl_2_, 1.0 g MgSO_4_·7H_2_O, 5.0 g sodium lactate, 1.0 g yeast extract, 50 ml Mohr’s salt solution (1 % w/v), and 1.0 ml resazurin (0.1 % w/v). The medium was degassed prior to the addition of 0.1 g l^−1^ Na-thioglycolate and 0.2 g l^−1^ cysteine-HCl under anoxic conditions (headspace 95% Ar, 5 % H_2_) and pH was adjusted to 8.1.

(iii) Modified Postgate sulfate enrichment medium [21] contained l^−1^ : 0.5 g K_2_HPO_4_, 1.0 g NH_4_Cl, 0.1 g CaCl_2_, 2.0 g MgSO_4_·7H_2_O, 2.0 g sodium lactate, 1.0 g yeast extract, 0.1 g ascorbic acid, 0.5 g FeSO_4_·7H_2_O, and 1.0 ml resazurin (0.1 % w/v). The medium was degassed prior to the addition of 0.1 g l^−1^ Na-thioglycolate and 0.2 g l^−1^ cysteine-HCl under anoxic conditions (headspace 95% Ar, 5 % H_2_) and pH was adjusted to 8.0.

(iv) Modified API medium [22] contained l^−1^ : 0.01 g K_2_HPO_4_, 10.0 g NaCl, 0.2 g MgSO _4_·7H_2_O, 3.5 g sodium lactate, 1.0 g yeast extract, 0.1 g ascorbic acid, 20 ml Mohr’s salt solution (1 % w/v) and 1.0 ml resazurin (0.1 % w/v). The medium was degassed prior to the addition of 0.2 g l^−1^ cysteine-HCl under anoxic conditions (headspace 95% Ar, 5 % H_2_) and pH was adjusted to 8.0.

(v) Filter sterile formation water with the addition of 0.5 g sodium acetate, 0.5 g K_2_HPO_4_, 1.0 g NH_4_Cl and 1.0 ml resazurin (0.1 % w/v). Dissolved organic carbon present in the formation water served as the carbon source. The medium was degassed prior to the addition of 0.2 g l^−1^ cysteine-HCl under anoxic conditions (headspace 95% Ar, 5 % H_2_).

### Enrichment and isolation method

Cultures were established anoxically in a Coy Anaerobic Chamber filled with ~95% argon, 2–3% nitrogen and 1–2% hydrogen (Coy Laboratory Products, MI, USA). Each culture was established in 200 ml borosilicate glass serum vials containing 50 ml of the various media described above with 100 µl of Surat Basin inoculum. Cultures were sealed with butyl rubber stoppers under the anerobic chamber atmosphere. After 2 weeks incubation at 30 °C in the dark without shaking, cultures were serially diluted 1 : 10 four times to a dilution of 1 : 10 000 in their respective media. After a further 2 week incubation under the conditions stated above, 16S rRNA sequencing was used to look for media in which *Desulfuromonas* sp. ‘CSMB_57’ was enriched. Subsequent serial dilutions were performed to extinction in order to remove contaminating taxa.

### DNA extraction and 16s rRNA sequencing

In order to confirm the presence of the target taxon in the enrichment culture and its abundance, subsamples of the enrichment culture were filtered through a 0.01 µm VVDF filter (Merck Millipore, Bayswater) and processed using the PowerSoil DNA Isolation Kit (MO Laboratories, USA) as per the manufacturer’s instructions with a modification to bead beating, which was performed on a FastPrep-24 (MP Biomedicals) for 40 s at 6 ms^−1^. PCR amplification was carried out with the Earth Microbiome Project (EMP) universal bacterial and archaeal primers 515F (5′-GTGCCAGCMGCCGCGGTAA-3′) and 806R (5′-GGACTACHVGGGTWTCTAAT-3′) [23]. Phusion High-Fidelity DNA Polymerase (New England Biolabs, Thermo Scientific) was used for the PCR reactions using the following cycle protocol: 94 °C for 3 mins, 30 × (94 °C for 45 s, 50 °C for 1 min, 72 °C for 1 min 30 s), 72 °C for 10 mins. Amplified PCR products were sent to Mr. DNA (TX, USA) for 16S rRNA sequencing.

### Genome sequencing, assembly, and annotation

DNA extraction was carried out using the PowerSoil DNA Isolation Kit (MO laboratories, USA) with a modification to the bead beating step, which was performed on a FastPrep-24 (MP Biomedicals) for 40 s at 6 ms^−1^. The DNA concentration was quantified using a Qubit dsDNA HS Assay Kit (Thermo Fisher Scientific, USA). The DNA library was carried out using the Nextera XT DNA Library Preparation Kit with the Nextera XT Index Kit (Illumina, USA). The volume and concentration of the DNA used were 5 µl and 0.2 ng µl^−1^, respectively. The resultant library was sequenced on a HiSeq2500 150 bp paired-end read length (Macrogen, South Korea). All procedures were carried out as per manufacturer’s instructions unless otherwise noted.

The genomic sequence data was error-corrected using Blue v2.1.4 [24] and then assembled using SPAdes v3.13.2 [25] with a target depth of 75 with the in-built error correction disabled. The genome assembly was manually curated by examining contig breaks, determining the cause and, where possible, gaps between the contig breaks were bridged. The resulting contigs were annotated using Prokka v1.14.5 [26] and a full length 16S rRNA sequence was reconstructed using Kelpie v2.0.11 [27]. In order to directly compare genes from the taxa described here and ‘*Candidatus* Desulfuromonas subbituminosa’ this taxon was downloaded from IMG/M [28] (IMG genome ID: 2603880216) and was also subjected to gene calling using Prokka v.1.14.5.

Genomes, contigs, predicted genes, and amino acid sequences were submitted to a number of tools to further explore the genetic potential of the genome. These tools included CheckM v1.1.3 to assess the quality of the assembled genome [29], BlastKOALA to map metabolic KEGG pathways [30], TransportDB v2.0 to identify membrane transport proteins [31], dbCAN for identification of enzymes involved in carbohydrate utilization [32], CRISPRFinder to reveal clustered regularly interspaced short palindromic repeats (CRISPR) [33], ISsaga to identify insertion-sequence (IS) elements [34], and antiSMASH to identify secondary metabolite biosynthesis gene clusters [35]. In order to facilitate comparisons with ‘*Ca*. Desulfuromonas subbituminosa*’,* genomic data from this taxon was also included in these analyses.

### Phylogenetics

The 16S rRNA phylogenetic tree was generated using mega11 [36] from 17 nucleotide sequences. The 16S rRNA sequences were aligned using muscle with the cluster method set to neighbour-joining [37]. The neighbour-joining phylogenetic tree was then constructed with a bootstrap value of 1000 [38] using the *p*-distance model [39] with complete deletion for the gaps and missing data treatment. For whole-genome comparison, the average nucleotide identity (ANI) of 16 genome sequences were calculated using the Orthologous Average Nucleotide Identity Tool (OAT) using the original ANI calculation [40]. The ANI output matrix was reconstructed into a tree using the neighbour-joining method [37] and converted to Newick format [41] prior to phylogenetic tree visualization with mega11 [36]. Default parameters were used unless otherwise noted.

## Results

### Enrichment and isolation

Through repeated serial dilutions, an enrichment culture was established using the modified basal sulphur reducing bacteria enrichment media. In order to confirm the identity of the target taxon, the 16S rRNA sequence from this enrichment culture was sequenced, identified and compared to the 16S rRNA sequence of the target taxon ‘CSMB_57’ [9] and was found to be 100% identical across 245 bp. Hereafter, the taxon is referred to as *Desulfuromonas* sp. ‘CSMB_57’. No growth of *Desulfuromonas* sp. ‘CSMB_57’ was detected in modified Baars, Postgate, API, or Formation Water media.

### 16s rRNA reconstruction from the genome and blast searches for *Desulfuromonas* sp. ‘CSMB_57’ in other environments

A full-length 16S rRNA sequence was reconstructed using Kelpie v2.0.11 [27]. blastn searches of the full-length sequence revealed 15 16S rRNA sequences with greater than 99% identity to *Desulfuromonas* sp. ‘CSMB_57’. Of these, the majority (10) were isolated from subsurface environments such as oil-field produced water, natural gas field, deep sedimentary aquifers, oil sands, underground natural gas storage, deep coal-seam groundwater, mud volcano and oil reservoirs (Table 1), while the remainder were from digestors. No strains were detected in commonly examined anoxic habitats such as animal digestive tracts, swampy environments, marine or freshwater sediments.

**Table 1.** Closely related taxa from GenBank with a blast search of >99% 16S rRNA identity match to *Desulfuromonas* sp. ‘CSMB_57’ from subsurface environments*

Accession no.	Percent ID	Location	Isolation source	Reference
KJ877716	100.00	China	Oil-field produced water	Liu and Shi 2014 – unpublished
AB701661	100.00	Japan	Natural gas field	Mayumi and Nakajima 2012 – unpublished
LC214865	100.00	Japan	Deep sedimentary aquifer	Katayama et al., 2017 – unpublished
EU522642	100.00	Canada	Oil sands tailings enrichment culture	[61]
GU339468	99.69	France	Underground natural gas storage	[62]
AB294283	99.69	Japan	Deep coal seam groundwater	[10]
JQ245693	99.58	Taiwan	Mud volcano	[63]
AY570613	99.39	Canada	Oil reservoir	[64]
AY570628	99.28	Canada	Oil reservoir	[64]
JQ088432	99.18	China	Crude oil reservoir	[65]

"*"Five other accessions matching ‘CSMB_57’ with high identity (>99 %) were also retrieved from anaerobic digestors (accession numbers: MH734878, MN414343, MK637487, AY692042 and MN434992).

**Fig. 1.**
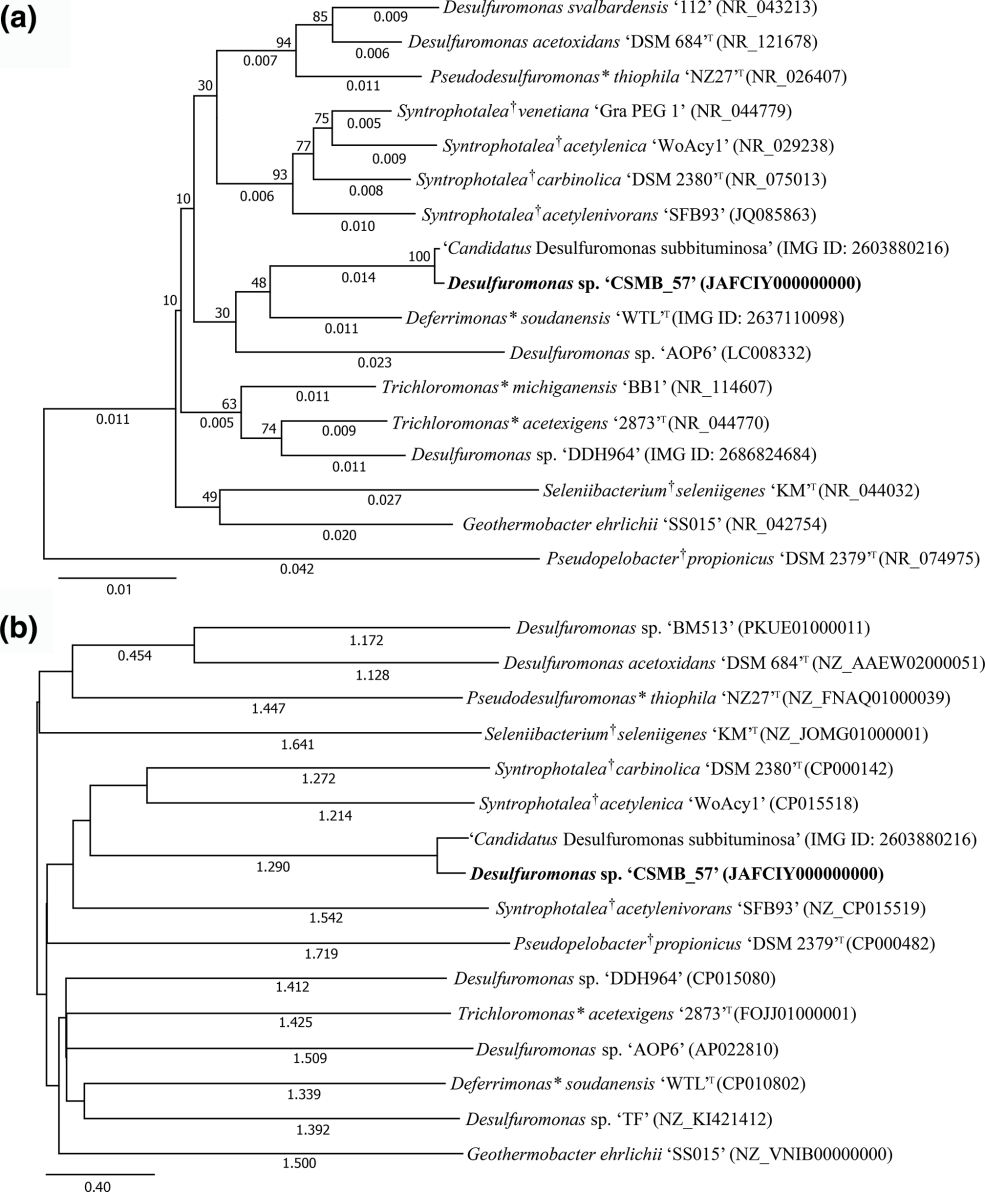
Phylogenetic trees were constructed based on (a) 16S rRNA and (b) whole-genome ANI. For the 16S rRNA phylogenetic tree, the evolutionary history was inferred using the neighbour-joining method [37]. The optimal tree is shown. The percentage of replicate trees in which the associated taxa clustered together in the bootstrap test (1000 replicates) are shown next to the branches [38]. The tree is drawn to scale, with branch lengths ≥0.005 shown in the same units as those of the evolutionary distances used to infer the phylogenetic tree. The evolutionary distances were computed using the *p*-distance method [39] and are in the units of the number of base differences per site. The 16S rRNA analysis involved 17 nucleotide sequences. All positions containing gaps and missing data were eliminated (complete deletion option). There were a total of 868 positions in the final dataset. Evolutionary analyses were conducted in mega11 [36]. *Pseudopelobacter propionicus* ‘DSM2379’^T^ is an outgroup in the 16S rRNA phylogenetic analysis. For the whole-genome ANI phylogenetic tree, the ANI between the 16 genomes were calculated using the Orthologous Average Nucleotide Identity Tool (OAT) [40]. The ANI output matrix was reconstructed into a tree using the neighbour-joining method [37] and converted to Newick format [41] prior to phylogenetic tree visualization with mega11 [36]. The tree is drawn to scale, with branch lengths ≥0.4 shown in the same units as those of the evolutionary distances used to infer the phylogenetic tree. Default parameters were used unless otherwise noted. Several taxa have been recently reclassified with * and † denoting taxa that were previously *Desulfuromonas* and *Pelobacter *, respectively.

In addition to taxa reported from environmental surveys, there were 15 related microbes with assembled genomes *Desulfuromonas acetoxidans* ‘DSM684’^T^, *Desulfuromonas* sp. ‘DDH964’, *Desulfuromonas* sp. ‘AOP6’, *Desulfuromonas* sp. ‘BM513’, *Desulfuromons* sp. ‘TF’, ‘*Ca*. Desulfuromonas subbituminosa’, *Deferrimonas soudanensis* ‘WTL’^T^, *Trichloromonas acetexigens* ‘2873’^T^, *Pseudodesulfuromonas thiophila* ‘NZ27’^T^, *Syntrophotalea carbinolica* ‘DSM2380’^T^, *Syntrophotalea acetylenica* ‘DSM3247’, *Syntrophotalea acetylenivorans* ‘SFB93’, *Pseudopelobacter propionicus* ‘DSM2379’^T^, *Seleniibacterium seleniigenes* ‘KM’^T^, and *Geothermobacter ehrlichii* ‘SS015’ (Table 2). The genome size and G+C content for these microbes ranged from 2.71 Mb to 5.09 Mb and 51.8–62.2%, respectively. These genomes were retrieved from GenBank and IMG/M databases and used in the phylogenetic analyses (Fig. 1).

**Table 2.** Summary statistics of genomes used for comparison

Genome	Genome size (Mb)	GC content (%)	Average contig length	N50 size measure	Isolation source	Accession no.	Ref
* Trichloromonas acetexigens * '2873'^T^	3.68	60.3	89 832	301 105	Digester sludge, sewage plant	FOJJ01000001	[66]
* Desulfuromonas acetoxidans * 'DSM 684'^T^	3.83	51.8	75 065	195 317	Sulfide rich seawater	NZ_AAEW02000051	i.
* Desulfuromonas* sp. ‘AOP6’	3.27	56.4	–	–	Subseafloor sediment	AP022810	[17]
* Desulfuromonas* sp. ‘BM513’	3.12	52.5	16 757	25 365	Estuary sediment	PKUE01000011	[67]
* Desulfuromonas* sp. ‘CSMB_57’	3.14	59.9	33 445	130 442	Coal seam	JAFCIY000000000	ii.
* Desulfuromonas* sp. ‘DDH964’	3.92	62.2	–	–	Deep subsurface brine	CP015080	iii.
* Desulfuromonas soudanensis * ‘WTL’^T^	3.96	61.2	–	–	Deep subsurface brine	CP010802	[68]
‘*Candidatus* Desulfuromonas subbituminosa’	2.71	60.3	150 292	498 748	Coal seam	2603880216*	iv.
* Desulfuromonas* sp. ‘TF’	4.40	58.6	258 985	394 359	Tidal flat	NZ_KI421412	[69]
*Pseudo-desulfuromonas thiophila* 'NZ27'^T^	2.79	60.7	71 537	111 360	Sediment	NZ_FNAQ01000039	v.
* Geothermobacter ehrlichii * 'SS015'	3.24	61.9	98 059	228 767	Pacific Ocean: axial seamount	NZ_VNIB00000000	vi.
* Syntrophotalea acetylenica * ‘WoAcy1’	3.18	57.4	–	–	Sediment	CP015518	[70]
* Syntrophotalea acetylenivorans * *‘*SFB93’	3.22	53.4	–	–	Intertidal sediment	NZ_CP015519	[70]
* Syntrophotalea carbinolica * 'DSM 2380'^T^	3.67	55.1	–	–	Anoxic mud	CP000142	[57]
* Pseudopelobacter propionicus * 'DSM 2379'^T^	4.01	59.0	–	–	Sediment	CP000482	i.
* Seleniibacterium seleniigenes * 'KM'^T^	5.09	54.1	848 062	3 076 292	Sediment	NZ_JOMG01000001	vii.

Whole-genomes are indicated by ‘-”. Asterisk (*) indicates IMG genome ID. Type species are noted by ‘^T^’.Additional references: i. Copeland *et al*. (unpublished), ii. this study, iii. Badalamenti and Bond (unpublished), iv. Robbins and Tyson (unpublished), v. Varghese (direct submission), vi. Goeker (unpublished), vii. Bini *et al*. (direct submission).

### Genome assembly and quality assessment

Genome assemblyof *Desulfuromonas sp*. ‘CSMB_57’ resulted in a draft genome of 3.14 Mb comprising 94 contigs (>200 bp), with a mean contig length of 33 445 bp, an N50 of 130 442 bp, and an average G+C content of 59.9%. CheckM v1.1.3 [29] was used to assess the quality of the assembled genome, which estimated the completeness and contamination to be 98.71% and 0.65%, respectively. Additional genomic information is shown in Table 2. The contigs for the *Desulfuromonas* sp. ‘CSMB_57’ genome were submitted to GenBank under the accession number JAFCIY000000000.

### 16s rRNA and genomic phylogenetic analyses

Genomic and 16S rRNA sequences of related taxa to ‘CSMB_57’ retrieved from GenBank and IMG/M databases were used for phylogenetic analyses (Fig. 1). The most closely related taxon to *Desulfuromonas* sp. ‘CSMB_57’ was ‘*Ca*. D. subbituminosa’ in both 16S rRNA (99.8% identity) and ANI (97.8% identity) comparisons. For the 16S rRNA phylogenetic analysis, ‘CSMB_57’ and ‘*Ca*. D. subbituminosa’ clustered together with *Deferrimonas soudanensis* ‘WTL’^T^ and *Desulfuromonas* sp. ‘AOP6’ forming a sister branch (Fig. 1a). In the genomic based phylogeny *Desulfuromonas* sp. ‘CSMB_57’ and ‘*Ca*. D. subbituminosa’ remained clustered together, however, they were clustered with different taxa with their nearest neighbours being the three *Syntrophotales* species: *S. acetylenica *, *S. acetylenivorans* and *S. carbinolica* (Fig. 1b).

### Gene calling and annotation

Prokka v1.14.5 [26] revealed the *Desulfuromonas* sp. ‘CSMB_57’ genome contained 2820 ORFs with 1226 hypothetical proteins, 48 tRNAs, two rRNAs, and one tmRNA (Data S1, available with the online version of this article).

### CRISPRs

CRISPRFinder [33] revealed one CRISPR gene array in *Desulfuromonas* sp. ‘CSMB_57’ with a direct repeats (DR) length of 32 bp with 108 spacers (Data S2).

### Database for automated carbohydrate-active enzyme annotation (dbCAN)

**Fig. 2.**
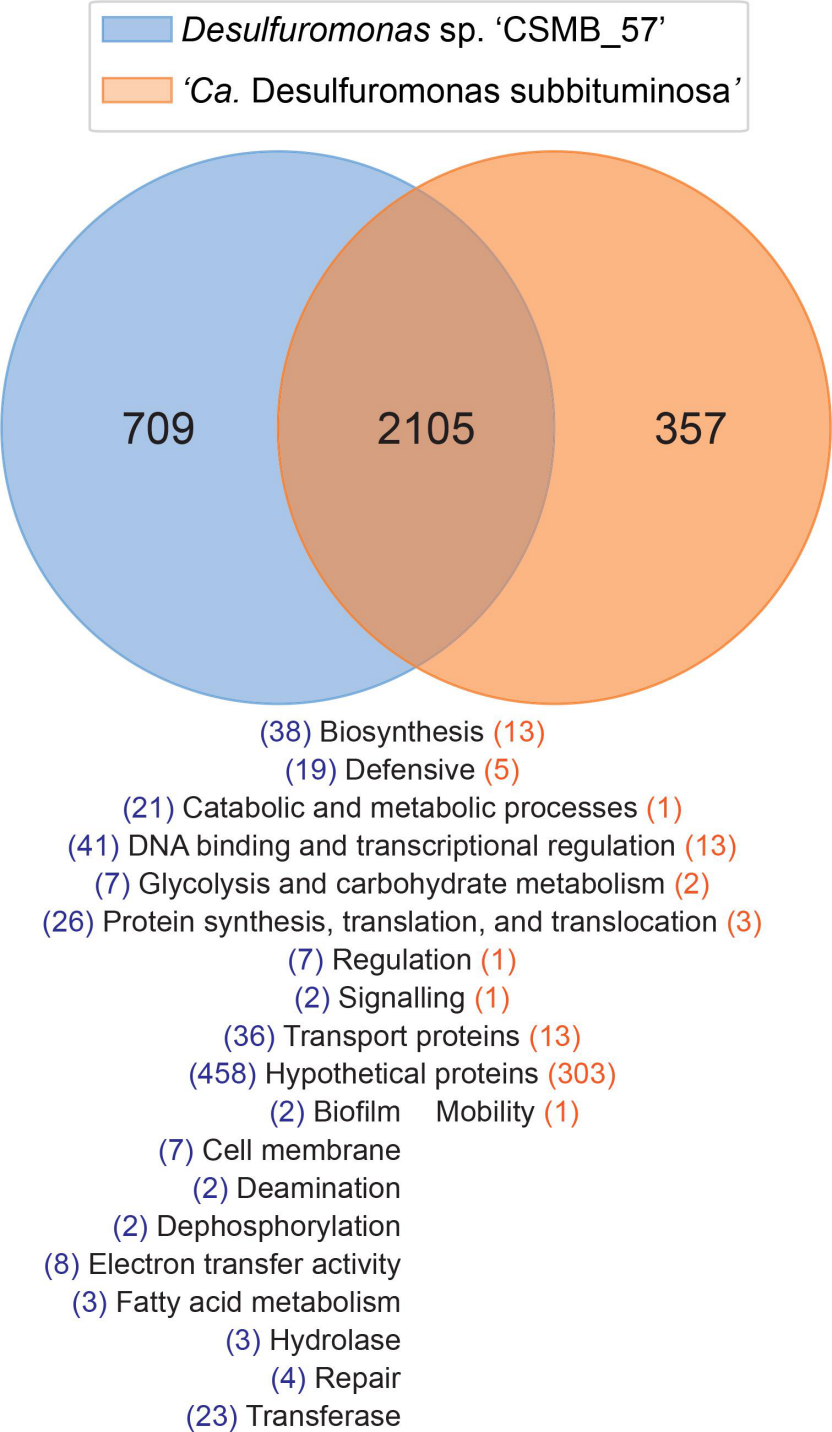
Venn diagram showing the shared and unique genes between *Desulfuromonas* sp. ‘CSMB_57’ (blue) and ‘*Candidatus* Desulfuromonas subbituminosa’ (orange). A summary of the functions of differing genes are listed below with further details in Data S3.

Fifty-eight carbohydrate active enzymes were predicted by dbCAN [32], of which nine contained signal peptides (Fig. 2). In summary, ‘CSMB_57’ contained three genes from the auxiliary activities (AA) family, one gene from the carbohydrate-binding modules (CBM) family, three genes from the carbohydrate esterase (CE) family, 16 genes from the glycoside hydrolase (GH) family, and two genes from the polysaccharide lyase (PL) family. Of the nine genes that had signal peptides, five genes were from the GH family, and the other four were from the AA, PL, CE, and glycosyltransferase (GT) families (Data S2).

### TransportDB

The TransportDB v2.0 [31] revealed a total of 261 ORFs coding for transport proteins with 87 ORFs associated with ABC transporters, 19 ORFs coding F-ATPases, 14 ORFs associated with the solute:sodium symporter family, 11 ORFs that code for major facilitator superfamily (MFS) membrane transporter proteins, another 11 ORFs for P-ATPases, seven ORFs coding the resistance-nodulation-division (RND) family transporters, six ORFs coding the type III (virulence-related) secretory pathway (IIISP) family, as well as another 106 ORFs spread across 50 other transport families (for full list see Data S2).

### The antibiotics and secondary metabolite analysis shell (antiSMASH) tool

The antiSMASH tool [35] was used to determine the number of antibiotic and secondary metabolite biosynthesis gene clusters in the ‘CSMB_57’ genome. A total of six secondary metabolite regions were identified: two terpenes, a phosphonate, a betalactone, a ribosomally synthesized and post-translationally modified peptide product (RiPP)-like, and a RiPP recognition element (RRE)-containing region (Data S2).

### Insertion sequence semi-automatic genomic annotation (ISsaga) tool

The ISsaga tool [34] was used to identify IS transposase elements in the ‘CSMB_57’ genome. A total of 16 predicted insertion elements were identified in the ‘CSMB_57’ genome.

### Comparisons between *Desulfuromonas* sp. ‘CSMB_57’ and ‘*a*. Desulfuromonas subbituminosa’

In total, 2105 genes (983 hypothetical and 1122 identified), were shared between the taxa with *Desulfuromonas* sp. ‘CSMB_57’ having 709 unique gene clusters (458 hypothetical and 251 identified) while ‘*Ca*. D. subbituminosa’ contained 357 unique gene clusters (303 hypothetical and 54 classified) (Fig. 2). Details of the putative functions of these genes are shown in Data S3.

## Discussion

The aim of the present study was to isolate ‘CSMB_57’ the most ubiquitous and abundant bacterial taxon in the eastern Australian coal seams (Bowen, Sydney and Surat Basins). Numerous studies have previously detected this taxon, for instance, it was first observed in the Ishikari Basin in Japan by Shimizu and co-workers in 2007 [10]. Since then, it has been observed in virtually all studies of eastern Australian coal seams associated with methane gas production [9]. Despite this ubiquity, its role is not well understood.

In the present study, a modified version of Pfennig and Biebl’s medium [15] was used to generate a highly enriched culture of *Desulfuromonas* sp. ‘CSMB_57’ for the first time through a dilution-to-extinction procedure. It is noteworthy that attempts to culture this taxon using solid media techniques, such as the roll-tube method or agar deeps, as well as using elemental sulphur in liquid media were unsuccessful. In this study, on the failure to enrich the target taxon using elemental sulphur, the authors utilized a polysulfide solution in the liquid medium, which greatly enhanced the enrichment of ‘CSMB_57’. This polysulfide solution was initially developed by Pfenning and Biebl in their 1976 study for agar deeps. The polysulfide solution – rather than elemental sulphur – was used as it improves the distribution of sulphur in solid media and, presumably, in liquid media it also enhances sulphur availability as ‘CSMB_57’ was successfully enriched. In parallel, dilutions of the same formation water in media containing sulphate (Baars, Postgate and API), did not result in growth of the taxon suggesting that ‘CSMB_57’, like other *Desulfuromonas* species, cannot use sulphate as an electron acceptor [16, 42]. blast analyses of the full-length 16S rRNA from the resultant culture revealed that all recorded natural occurrences of the organism were associated with the subsurface, though it was also detected in a small number of non-natural habitats, such as anaerobic digestors. For instance, 16S genes with >99 % identity to ‘CSMB_57’, were detected in oil reservoirs, natural gas fields, deep aquifers, oil sands tailings and in a mud volcano located in Canada, China, France, Japan and Taiwan (Table 1). Interestingly, there are no records of this taxon from other common, sulphur-rich environments such as mangroves, marine or freshwater sediments. This suggests that ‘CSMB_57’ may be a subsurface specialist with a wide distribution in both the northern and southern hemisphere. It is noteworthy that most of the environments where ‘CSMB_57’ was detected were also associated with hydrocarbons (e.g. oils, tar or coals), however, more sampling of the subsurface has been undertaken in association with fuel sources and this may represent something of a bias in the data. It would be valuable to examine hydrocarbon-free subsurface environments more completely to understand whether taxa closely related to ‘CSMB_57’ occur more broadly.

The 16S rRNA phylogenetic analysis conducted in this study supports the recent reclassifications of taxa within the Deltaproteobacteria class (see Waite *et al*. [12]) as the updated genera now cluster into well-defined groups (Fig. 1a). In particular, the previously polyphyletic genus *Pelobacter* has been revised and now consists of three separate genera, the *Syntrophotalea *, *Seleniibacterium* and *Pseudopelobacter* (Fig. 1a). The *Desulfuromonas* genus has also been revised with new genera, such as *Pseudodesulfuromonas *, *Deferrimonas* and *Trichloromonas* while *Desulfuromonas acetoxidans* ‘DSM 684’^T^ remains as the type species for the *Desulfuromonas* genus. In relation to *Desulfuromonas* sp. ‘CSMB_57’, it forms part of a well-supported clade of taxa that included its closest relatives: ‘*Ca*. Desulfuromonas subbituminosa’, *Deferrimonas soudanensis* ‘WTL’^T^ (the most closely related type species) and *Desulfuromonas* sp. ‘AOP6’ along with a sister clade that includes *Trichloromonas acetexigens* ‘2873’^T^, *Trichloromonas michiganensis* ‘BB1’, and *Desulfuromonas* sp. ‘DDH964’ (Fig. 1a).

In contrast to the 16S rRNA phylogeny, whole-genome comparisons were conducted using ANI, which revealed a different phylogenetic arrangement (Fig. 1b). It is noteworthy to mention that genome comparisons using ANI includes all coding regions in the genome and some of this may be horizontally acquired. This gives the whole-genome analysis increased resolution compared to the 16S rRNA analysis [43]. The whole-genome phylogenetic tree shows ‘CSMB_57’ grouping with *Syntrophotalea* spp. and is more distant from *Desulfuromonas* sp. ‘AOP6’ and *Deferrimonas soudanensis* suggesting that, at the genomic level, these taxa are either more distantly related to ‘CSMB_57’ or have obtained significant genetic material laterally. This disparity is also evident when comparing the G+C content and genome size of the genomes that were investigated (Table 2). The *Syntrophotalea* genus has a much narrower G+C content range of 53.4–57.1% in comparison to the *Desulfuromonas* spp., which range from 51.8–62.2%. The differences between genome size across the *Desulfuromonas* genus is also quite large, ranging from 2.71 to 4.40 Mb, compared to the *Syntrophotalea* genus 3.18 Mb to 3.67 Mb (Table 2). It is important to note, however, that some of these *Desulfuromonas* species have yet to be mentioned for reclassification, such as, *D*. sp. ‘AOP6’, *D*. sp. ‘BM513’, *D*. sp. ‘TF’ and *D*. sp. ‘DDH964’, which might be classified as a *Trichloromonas* as it groups with *Trichloromonas acetexigens* ‘2873’^T^ in the 16S rRNA and whole-genome phylogenetic trees (Fig. 1). Comparing the genomic information of ‘CSMB_57’ with *Syntrophotalea* reveals that its genome size is similar (3.14 Mb), however, the G+C content is higher (59.9 %). Even though ‘CSMB_57’ and its close relative ‘*Ca*. D. subbituminosa’ form a defined clade with *Syntrophotalea* in the whole-genome tree (Fig. 1b), they do not appear to be part of the *Syntrophotalea* genus from a 16S rRNA perspective (Fig. 1a). Taking these genomic characteristics into consideration, these two taxa (*D*. sp. ‘CSMB_57’ and ‘*Ca*. D. subbituminosa’) may be part of a new genus and should be considered during the current reclassification of the Desulfuromonadales order [12, 14].

Regardless of whether 16S or genomic data is used to make phylogenetic inferences, the closest related taxon to ‘CSMB_57’ was ‘*Ca*. Desulfuromonas subbituminosa’. The two taxa share 99.8% identity in the full-length 16S rRNA, have an ANI of 97.8 %, and are likely conspecific, though the ‘*Ca*. D. subbituminosa’ draft genome is markedly smaller (0.43 Mb) than ‘CSMB_57’. Comparison of the gene annotations between ‘*Ca*. D. subbituminosa’ and ‘CSMB_57’ revealed that a number of genes with ecological relevance differed between the two taxa. The genome of ‘CSMB_57’, for instance, includes numerous additional genes involved in CRISPR utilization, which are absent from ‘*Ca*. D. subbituminosa’ and previous studies have indicated that numerous subsurface organisms have a large number of CRISPR arrays [44, 45]. Intriguingly, CRISPRFinder analyses presented here revealed a broadly similar number of CRISPR arrays were also detected in ‘CSMB_57’ and ‘*Ca*. D. subbituminosa’. The absence of these additional genes in ‘*Ca*. D. subbituminosa’ may indicate differences between strains of this taxon, be an artefact of the metagenomic assembly, or represent genes that the ‘CSMB_57’ strain has acquired laterally. In a similar fashion, the ‘CSMB_57’ genome encodes a number of antibiotic resistance related genes that are absent from ‘*Ca*. D. subbituminosa’. It may be that these genes have also been laterally acquired by ‘CSMB_57’ and may have been part of the parent metagenome from which ‘*Ca*. D. subbituminosa’ was assembled but were not included in the metagenomic assembly due to differences in their short *k*-mer frequencies that were used to assign contigs to bins. Further work on the taxon to help clarify the core- and pan-genome would assist in our understanding of the broad capabilities of the taxon as a whole.

One key goal of this research was to improve our understanding of the role that ‘CSMB_57’ may play in subsurface coal seams. It has been suggested that Deltaproteobacteria in the coal seams likely form syntrophic relationships as they have been shown to be associated with methanogens and/or acetogens [3, 46–53]. The suggestion is presumably based on the well-studied relationships between *Desulfovibrio* spp. [48, 49] or *Syntrophotalea* spp. [50–54] with methanogens and/or acetogenic taxa. In 1984 and 1985, for example, Schink [52, 53] demonstrated that *Syntrophotaela carbinolica* and *Syntrophotaela acetylenica* (previously *Pelobacter carbinolicus* and *Pelobacter acetylenicus *, respectively), close relatives of ‘CSMB_57’ (Fig. 1), were capable of syntrophic growth with hydrogen scavenging microbes (either *Methanospirillum hungatei* or *Acetobacterium woodii *) via interspecies hydrogen transfer (IHT) [50]. In co-culture, these *Syntrophotalea* spp. mainly converted primary alcohols to H_2_ (and presumably CO_2_) and acetate when *Methanospirillum hungatei* was the syntrophic partner, and produced only acetate when *Acetobacterium woodii* was the syntrophic partner [52, 53]. This indicates that in these gnotobiotic cultures, *Syntrophotalea* spp. are capable of oxidizing primary alcohols, which produces H_2_, CO_2_, and acetate while engaged in syntrophy with hydrogen scavenging microbes.

Evidence for *Desulfuromonas sensu stricto* being involved in syntrophy, however, is limited. One study by Biebl and Pfennig in 1978 [55], for instance, demonstrated *Desulfuromonas* formed syntrophy with phototrophic green bacteria (* Chlorobium* or *Prosthecochloris *). In these relationships the reduction of sulphur was coupled with the degradation of ethanol to produce hydrogen sulfide and CO_2_ which, in turn, was utilized by the phototrophic green bacteria partners. In the coal-seam environment, however, there is no light for phototrophy and the coal seams and formation waters of the environments in which ‘CSMB_57’ has been observed are very low in sulphur [18]. Recently, a study by Guo and co-workers in 2021 [17] conducted an in-depth genomic analysis of five species closely related to ‘CSMB_57’: *Desulfuromonas* sp. ‘AOP6’, *Trichloromonas acetexigens* ‘2873’^T^, *Deferrimonas soudanensis* ‘WTL’^T^, *Desulfuromonas* sp. ‘DDH964’ and *Desulfuromonas* sp. ‘TF’. The aim of this study was to identify specific genomic signatures between Fe (III) oxide stimulated taxa and anode-stimulated taxa. The study identified the main genomic signatures of the Fe (III) oxide stimulated taxon ‘AOP6’ were the possession of the flagellar biosynthesis gene cluster as well as diverse abundant genes associated with the chemotaxis sensory systems (40 *che* genes across nine types and 14 *mcp* genes) and *c*-type cytochromes (28 othologous groups). Conversely, the anode-stimulated taxa 'WTL’ and ‘DDH964’ lacked the flagellar biosynthesis cluster, and contained less diverse chemotaxis sensory systems (an average of 35 *che* genes across seven types and 13 *mcp* genes) and *c*-type cytochromes (24 and 19 orthologous groups, respectively), however, these taxa had increased oxygen resistance and transposable gene elements, which may provide the capability for genomic rearrangement.

Initial comparisons between ‘CSMB_57’ and the previously mentioned taxa revealed that, although there is a similarity with ‘AOP6’ as ‘CSMB_57’ has 24 genes associated with flagellar biosynthesis (Data S1), ‘CSMB_57’ contains fewer chemotaxis sensory genes (17 *che* genes across nine types and 10 *mcp* genes) and *c*-type cytochromes (three genes) compared to the other taxa. The chemotaxis sensory genes are typically observed to be in close proximity to flagellar clusters, which suggests that they might be related to flagellar-based motility that allows microbes to respond to environmental stimuli [17]. Therefore, the presence of a flagellar biosynthesis cluster in ‘CSMB_57’ and a diverse, albeit low number, of chemotaxis genes suggests that ‘CSMB_57’ might be capable of sensing its surrounding environment and generate motility in response. The low number of *c*-type cytochromes in ‘CSMB_57’ is unusual for a *Desulfuromonas* species as they are used for electron transport during metal oxide and electrode respirations, one of the key characteristics of *Desulfuromonas* species. Whether or not ‘CSMB_57’ is capable of reducing Fe (III) is, however, still unknown and future studies would be required to investigate this further.

Despite ‘CSMB_57’ being initially assigned as a *Desulfuromonas* species, its genomic features are more similar to that of *Syntrophotalea *, i.e. the number of *c*-type cytochromes [56] and chemotaxis sensory genes [57] in *Syntrophotalea carbinolica* (previously *Pelobacter carbinolicus *) are comparable to ‘CSMB_57’. These genomic characteristics, together with the whole-genome phylogenetic tree (Fig. 1b), indicates that the metabolic capability of ‘CSMB_57’ may be more similar to *Syntrophotalea* spp. as opposed to *Desulfuromonas* spp. If this is the case, then ‘CSMB_57’ may have the capacity for syntrophy with methanogens and/or acetogens in a similar way as described with *S. carbinolica* and *S. aceytenlica* [50–54]. The molecular mechanism that drives these types of syntrophic relationships in methanogenic environments involve interspecies electron transfer such as IHT, interspecies formate transfer and direct interspecies electron transfer (DIET) via pili and/or outer membrane cytochromes [50]. During syntrophic growth with methanogens, *S. carbinolica* and *S. acetylenica*typically utilize hydrogenase enzymes for IHT, which is coupled with the oxidation of primary alcohols via alcohol dehydrogenases [50–54]. There are also a number of other enzymes which are associated with this process, namely aldehyde dehydrogenase, phosphate acetyltransferase, and acetate kinase [53, 54]. Genes encodoing these enzymes were detected in ‘CSMB_57’: alcohol dehydrogenase *yqhD*; aldehyde dehydrogenase *ALDH*; acetate kinase *ackA*; and phosphate acetyltransferase *pta* (Supplemetary Data 1). Due to the similarities mentioned above between ‘CSMB_57’ and *Syntrophotalea* spp. it may be possible that ‘CSMB_57’ is able to oxidize ethanol while in a syntrophic relationship with methanogens. Although it is unknown how much ethanol is produced in coal seams, it is a product of primary fermentation of organic matter and may be available for subsequent syntrophic fermentation [8]. Other potential organic substrates for syntrophic fermentation are various types of organic acids, which have been observed to be produced during the anaerobic biodegradation of coal to methane under laboratory conditions [58]. In addition, the numerical abundance of methanogens, in particular *Methanobacterium* sp. (‘CSMB_178’) and *Methanocalculus* sp. (‘CSMB_203’), and ‘CSMB_57’ in surveys of eastern Australian coal seams [9] compared with the relatively low abundance of acetogenic taxa suggests that ‘CSMB_57’ is likely more frequently involved in syntrophy with hydrogenotrophic methanogens rather than acetogens.

**Fig. 3.**
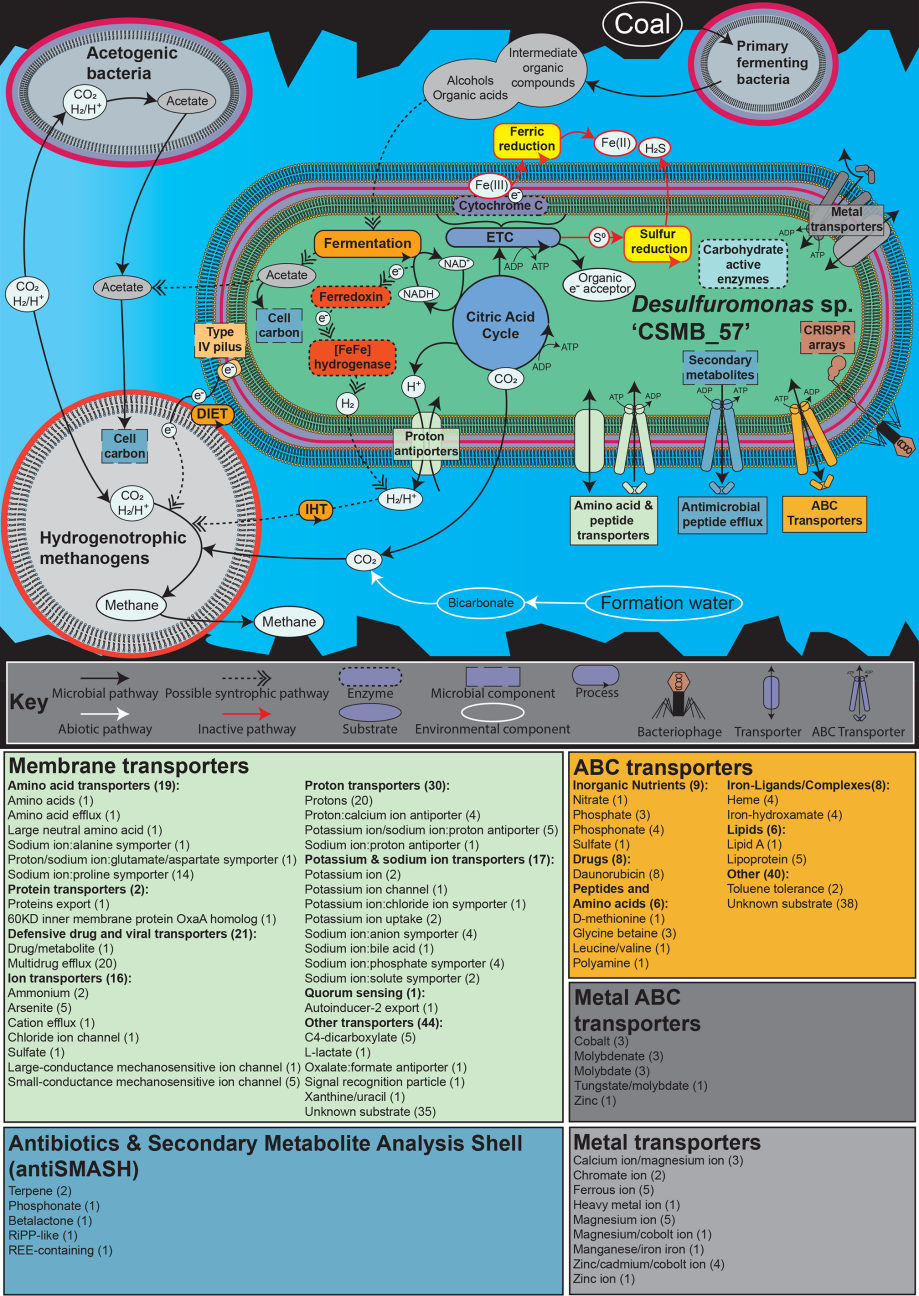
Ecological model depicting the metabolic processes and possible syntrophic relationships of *Desulfuromonas* sp. ‘CSMB_57’. Abbreviations: ABC, ATP-binding cassette; CRISPR, clustered regularly interspaced short palindromic repeats; DIET, direct interspecies electron transfer; ETC, electron transport chain; IHT, interspecies hydrogen transfer.

Using data presented here, a conceptual model of the role *Desulfuromonas* sp. ‘CSMB_57’ may play in the coal-seam environment was developed (Fig. 3). The conceptual model depicts primary fermenting bacteria catabolizing coal into intermediate organic compounds, which includes alcohols and organic acids. *Desulfuromonas* sp. ‘CSMB_57’ acts as the secondary fermenting bacteria as it syntrophically metabolizes alcohols and organic acids. The fermentation process involves the oxidation of NADH to NAD^+^ coupled with ferredoxin to produce reduced ferredoxin, which subsequently produces H_2_, catalysed by [FeFe] hydrogenase [59]. The final electron acceptor would involve an organic compound, possibly pyruvate, though this has yet to be determined. The main product generated through this anaerobic fermentation process is likely acetate with some being used for cell carbon while the excess is transported out of ‘CSMB_57’, which the methanogen might uptake and use for its own cell carbon. In addition to acetate, CO_2_ and H_2_ would be released into the surrounding environment as well. These substrates are possibly competed for between acetogenic bacteria and hydrogenotrophic methanogens, though as previously mentioned, due to the higher abundance of methanogens present in the eastern Australian coal seams, it is likely that the methanogens are the dominant hydrogen consumers. This consumption facilitates the syntrophic interaction between hydrogenotrophic methanogens and ‘CSMB_57’ as it enables the fermentation of organic substrates. This, in turn, provides the substrates for methanogenesis and is likely driven by DIET, which involves the transfer of electrons via the type-IV pilus, and/or IHT. Supporting such a conjecture are data that show the closely related taxon *D. acetoxidans* which uses type-IVa pili while in syntrophy with phototrophic bacteria [60]. Further evidence that may indicate pili involvement in this relationship are genes in ‘CSMB_57’ for a type-IV pilus (type-IV pilus biogenesis factor PilY1 and type-IV pilus biogenesis and competence protein PilQ) detected by the genome annotation. There are also indications that ‘CSMB_57’ may also have the capability to actively scavenge for environmental nutrients. A total of 252 transporters were identified in ‘CSMB_57’ using TransportDB v2.0 [31] of which 88 were ABC transporters. The high number of ABC transporters identified in ‘CSMB_57’, as well as the presence of chemotaxis sensory and motility genes, may be an indication that it is capable of scavenging substrates. It is important to note that the absence of sugar transporters and limited carbohydrate active enzymesindicate that, unlike other taxa recently isolated from the same environment that have a host of carbohydrate active enzymes [45], ‘CSMB_57’ is likely not involved in sugar uptake and biofilm recycling. Therefore, this type of scavenging strategy is likely an ancillary source of energy production and growth for ‘CSMB_57’. Additional components of ‘CSMB_57’ are also depicted in the conceptual model. The ferric and sulphur reduction pathways are shown, though are likely to be inactive in the coal-seam environment due to trace amounts of ferric iron and elemental sulphur. There are also defensive components present, namely CRISPR arrays, and antimicrobial metabolites and efflux pumps.

The present study successfully enriched the most abundant and ubiquitous bacterial taxon in eastern Australia coal seams. Based on its abundance and relatively limited catabolic potential it seems likely that *Desulfuromonas* sp. ‘CSMB_57’ is engaged in a syntrophic relationship with *Methanobacterium* or *Methanocalculus* species. The organic substrates that ‘CSMB_57’ may be utilizing during syntrophy are possibly alcohols and organic acids. Future work is required to experimentally demonstrate and confirm these potential syntrophic relationships and substrates through monitored gnotobiotic culturing. Similarly, work to better define the genetic variability within this taxon would be valuable to better understand the abilities of strains in the various basins of eastern Australia.
